# Validation of a prognostic scoring system for brain metastases with synchronous detection of primary cancers at initial consultation

**DOI:** 10.3389/fonc.2025.1617366

**Published:** 2025-11-17

**Authors:** Yohei Yamamoto, Gentoku Saito, Tomona Maetani, Yurika Terasawa, Naoki Kato, Takuya Ishii, Yasuharu Akasaki, Yuichi Murayama, Toshihide Tanaka

**Affiliations:** 1 Department of Neurosurgery, The Jikei University School of Medicine, Daisan Hospital, Tokyo, Japan; 2 Department of Neurosurgery, The Jikei University School of Medicine, Tokyo, Japan

**Keywords:** brain metastases (BM), synchronous brain metastases, prognostic factors (PF), prognostic scoring system, survival analysis

## Abstract

**Objective:**

To develop and validate a simple 0–7 point prognostic score for patients with synchronous brain metastases (s-BM) at initial consultation and to provide an exploratory comparison with metachronous BM (m-BM).

**Methods:**

We retrospectively analyzed 297 patients with BM (2014–2022): s-BM n=64 and m-BM n=233. The score uses five pre-treatment factors available at time-zero: age <70 y (1 point), KPS ≥70 (2 points), absence of extracranial metastases (ECM) (2 points), presence of a lung mass on imaging (1 point), and absence of a digestive-tract mass (1 point). Patients were stratified as Score A (0), B (1–4), and C (5–7). Discrimination and parsimony were assessed by Harrell’s C-index and AIC; proportional-hazards (PH) assumptions by global Schoenfeld test; time-dependent AUCs at 6/12 months were calculated; bootstrap (B = 500) provided optimism-corrected C-indices. (Synchronous was defined as ≤30 days between first ascertainment of primary tumor and BM; m-BM >30 days).

**Results:**

Median OS appeared similar for s-BM and m-BM in this cohort (5.2 *vs* 6.4 months; exploratory). In s-BM, KPS ≥70 and absence of ECM were independent predictors of longer survival. The proposed score produced stepwise separation across A/B/C. It showed the highest C-index (0.690) and lowest AIC (375.57) versus RPA, GPA, SIR, and BSBM, with no PH violations (global p=0.983). AUCs were 0.779 (6 mo) and 0.795 (12 mo); bootstrap-corrected C-index ≈0.691. A coefficient-weighted variant yielded a similar C-index (0.694) but a higher AIC (381.9).

**Conclusions:**

A five-factor, 1–2 point score usable at initial consultation provides superior discrimination and parsimony in s-BM compared with established systems. External, multicenter validation is warranted.

## Introduction

Brain metastases (BM) are considered to be the “terminal stage” of cancers due to their dismal prognosis. Multimodal therapeutics including surgical resection, chemoradiation therapy, and immunotherapy have contributed to extending median overall survival (mOS) and improving the quality of life for patients with cancer involving BM.

Considering the timing of initial diagnoses of BM along with primary lesions, BM could develop after the progression of the primary lesion [defined as metachronous BM (m-BM)] or BM develop simultaneously with or before treatment for the primary lesions [defined as synchronous BM (s-BM)]. So far, several prognostic parameters, including recursive partitioning analysis (RPA), graded prognostic assessment (GPA), score index for radiosurgery (SIR), and basic score for brain metastases (BSBM), have been utilized for validation for focal radiotherapy such as stereotactic radiosurgery (SRS) (1–4). In the era of combined immunotherapy of immune checkpoint inhibitors (ICIs), molecular target agents, and radiation therapy, aggressive treatment for BM, even under the co-existence of primary lesion and BM, might provide clinical benefit. Given that the genetic profile for driver mutations of cancer-related oncogenes and immune checkpoint molecules of PD-L1 with tumor mutation burden and microsatellite instability might stratify clinical outcomes in patients with the same primary cancers, we need a predictive prognostic biomarker for reflecting the clinical prognosis of patients with BM.

The purpose of this study was to explore several parameters extracted by multivariate analysis in a retrospective cohort of patients with s-BM and m-BM, to establish a novel predictive score based on these parameters for validation of multimodal treatment including molecular targeted drugs and ICIs. This novel scoring system might provide accurate prognostic prediction judged from the initial parameters to facilitate medical collaboration with other departments and to determine an appropriate sequential therapeutic schedule. We present a descriptive, exploratory comparison of s-BM and m-BM to contextualize our cohort, recognizing that the study is not designed or powered to test equivalence. Regardless of apparent similarity in survival, clinical decision-making at initial consultation differs fundamentally in s-BM; therefore, our primary objective was to develop an s-BM–specific prognostic score based on pre-treatment information.

## Materials and methods

### Study design and patients

We conducted a single-center retrospective cohort study of consecutive patients with brain metastases (BM) treated at The Jikei University School of Medicine Daisan Hospital between January 2014 and December 2022. Baseline demographic and clinical variables were recorded and are reported in the Results (**Table 1**); no descriptive counts are repeated in Methods.

**Table 1:** Clinical characteristics of synchronous and metachronous brain metastasis.

	Main factor	Sub factor	Synchronous	Metachronous	P value
Pretreatment factors	age	over 70	15	52	0.57
		under 70	49	181	
	sex	female	27	99	0.97
		male	37	134	
	symptomatology	asymptomatic	26	93	0.92
		symptomatic	38	140	
	KPS	under 60	35	125	0.88
		over 70	29	108	
	cyst lesion	(-)	45	161	0.85
		(+)	19	72	
	hematoma	(-)	63	226	0.53
		(+)	1	7	
	meningitis	(-)	57	203	0.68
		(+)	7	30	
	posterior fossa lesion	(-)	41	135	0.38
		(+)	23	98	
	extra cranial metastasis	(-)	24	122	0.04*
		(+)	40	111	
	Number of metastatic lesions	multiple	41	141	0.61
		single	23	92	
	lung origin	(-)	17	94	0.04*
		(+)	47	139	
	breast origin	(-)	62	203	0.03*
		(+)	2	30	
	digestive origin	(-)	56	200	0.73
		(+)	8	33	
	renal origin	(-)	63	223	0.31
		(+)	1	10	
	other origin	(-)	58	213	0.84
		(+)	6	20	
Therapeutic factors	surgery	(-)	45	206	<0.01*
		(+)	19	27	
	chemotherapy	(-)	21	24	<0.01*
		(+)	43	209	
	conventional chemotherapy	(-)	46	125	<0.01*
		(+)	18	108	
	Molecular targeted therapy	(-)	45	154	0.53
		(+)	19	79	
	ICI	(-)	56	203	0.94
		(+)	8	30	
	radiation	(-)	26	71	0.13
		(+)	38	162	
	SRS	(-)	38	138	0.98
		(+)	26	95	
	whole brain radiation	(-)	51	160	0.09
		(+)	13	73	
	neuro death	(-)	46	161	0.59
		(+)	10	43	

ICI, immune checkpoint inhibitor; KPS, Karnofsy performance status.

SRS, stereotactic radiosurgery; *P<0.05.

### Definitions

“Synchronous” BM (s-BM) was defined as an absolute interval ≤30 days between the first ascertainment of the primary tumor and the first ascertainment of BM, regardless of order; “metachronous” (m-BM) as >30 days. Ascertainment dates were the earliest imaging or histopathology confirmations. (We did not use “after start of primary therapy” as a criterion to avoid conflict with the time-window definition).

### Pretreatment variables and imaging surrogates

Five time-zero variables were prespecified: age, Karnofsky Performance Status (KPS), extracranial metastases (ECM), lung mass, and digestive-tract mass. Clinically interpretable cutoffs were set *a priori* (age <70 y; KPS ≥70). Because the score is intended for use at initial consultation, when histology may be unavailable, we used imaging-based surrogates for primary site when necessary: “lung mass” (discrete intrathoracic mass on chest radiograph/CT judged by the treating team to represent a presumptive lung primary) and “digestive mass” (discrete mass in the esophagus, stomach, intestine, pancreas, or hepatobiliary system on CT/US). These definitions used the earliest imaging before brain-directed therapy and were not retroactively altered by later pathology to avoid look-ahead/incorporation bias.

### Score construction

Variables significant in univariable Cox received 1 point; those retaining significance in a multivariable Cox model received 2 points, yielding a 0–7 total. As sensitivity analyses, we (i) derived a coefficient-weighted score from the multivariable β estimates and (ii) fitted a reduced score including only multivariable-significant factors; both were compared with the simple scheme using C-index and AIC (Supplement).

### Comparator systems

For external benchmarks, we implemented the original descriptions of RPA (1), GPA (2), SIR (3), and BSBM (4), using their published category definitions (canonical sources cited).

### Outcome

The primary endpoint was overall survival (OS) from initial consultation to death; patients alive at last contact were censored.

### Statistical analysis

Kaplan–Meier curves were compared with two-sided log-rank tests. Prognostic effects were estimated using Cox proportional-hazards models. Proportional-hazards (PH) assumptions were assessed by the Grambsch–Therneau test on Schoenfeld residuals (cox.zph) for global and variable-specific tests; diagnostic plots were inspected. To compare systems beyond hypothesis testing, we reported Harrell’s C-index (discrimination), Akaike information criterion (AIC) (parsimony), and time-dependent AUCs at 6 and 12 months (timeROC); for categorical systems (RPA, GPA, SIR, BSBM), discrimination was evaluated via the Cox linear predictor. Bootstrap internal validation (B = 500 resamples) provided optimism-corrected C-indices. Missing data were handled by complete-case analysis per model. All tests were two-sided with α = 0.05. Analyses were performed in R and EZR (based on R) (5).

## Results

### Comparison of mOS between s-BM and m-BM

**Figure 1:**
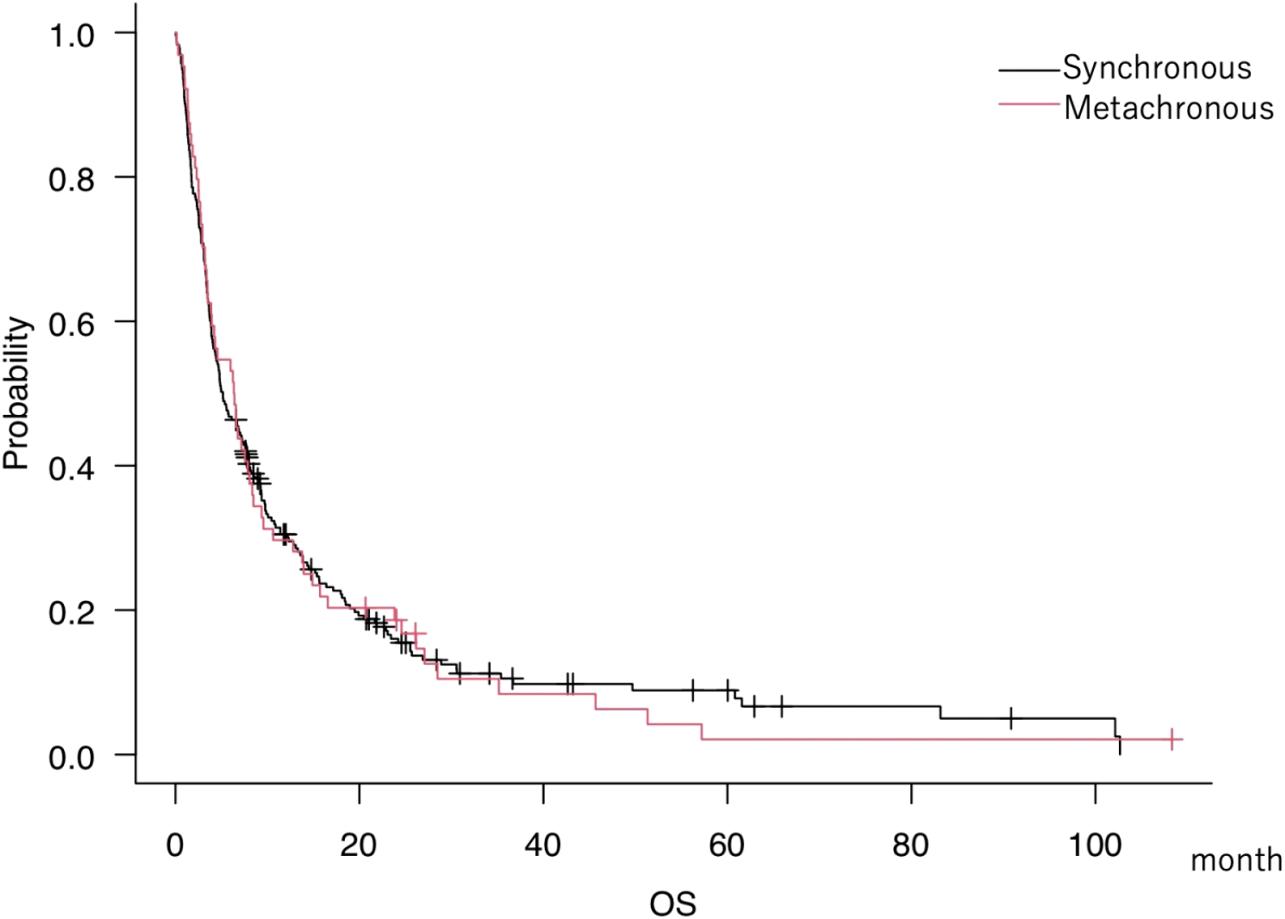
Kaplan–Meier overall survival (OS) curves for synchronous brain metastases (s-BM; n=64) and metachronous BM (m-BM; n=233) at initial consultation. OS was measured from initial consultation to death; tick marks indicate censoring. Medians with 95% CIs are shown (s-BM 5.2 months [4.1–7.3]**;** m-BM 6.4 months [3.5–8.0]**).** This two-group comparison is exploratory and not powered for equivalence/non-inferiority. Differences were assessed with a two-sided log-rank test**;** an unadjusted hazard ratio (Cox model) is reported in the Supplement for context. Abbreviations: OS, overall survival; s-BM, synchronous brain metastases; m-BM, metachronous brain metastases.

Median overall survival (mOS) was 5.2 months (95% CI, 4.1–7.3) for s-BM and 6.4 months (95% CI, 3.5–8.0) for m-BM. We did not detect a statistically significant difference between groups (**Figure 1**); this two-group comparison is exploratory and not powered for equivalence/non-inferiority. Our primary analyses focus on developing and validating the s-BM prognostic score. As pretreatment background, the s-BM group had more extracranial metastasis, more primary lung cancer, and fewer breast cancer cases, and in terms of treatment, surgery was more frequent and chemotherapy—especially conventional regimens—was less frequently administered in s-BM. Cohort characteristics.

We analyzed 297 patients overall (s-BM n=64; m-BM n=233). Key baseline features are summarized in **Table 1**, including age, sex, primary tumor sites, KPS, extracranial metastases, lesion characteristics, and initial treatments. Values are presented as median (IQR) or n (%) (**Table 1**).

### Pretreatment factors associated with survival in s-BM

In s-BM, univariable analyses identified age <70 years, KPS ≥70, absence of extracranial metastasis (ECM), presence of primary lung cancer, and absence of primary digestive cancer as factors associated with prolonged mOS (**Table 2**). In multivariable analysis including these five factors, KPS ≥70 and absence of ECM remained independently associated with longer mOS (**Table 3**).

**Table 2:** Univariate analysis of mean survival time in patients where synchronous and metachronous brain metastasi.

Factor	Synchronous cases				Metachronous cases			
	Number of patients	mOS	95% confidence interval	P value	Number of patients	mOS	95% confidence interval	P value
age<70	23	8.3	3.9-24.6	0.02*	95	5.2	4.0-8.9	0.96
age≧70	41	4.6	2.9-7.2		138	5.1	3.8-7.2	
KPS≦60	35	3.4	2.5-6.3	<0.01*	125	3.1	2.5-3.8	<0.01*
KPS≧70	29	9.4	6.2-14.9		108	13.1	9.8-15.6	
female	27	6.2	3.2-9.4	0.79	99	7.7	4.7-9.8	0.01*
male	37	6.4	3.5-13.8		134	4.3	3.4-5.5	
asymptomatic	26	5	2.5-7.6	0.38	93	9.9	7.9-13.8	<0.01*
symptomatic	38	6.6	3.5-10.6		140	3.9	3.1-4.8	
multiple lesions	41	6.6	2.8-8.3	0.86	141	4	3.4-5.2	<0.01*
single lesion	23	6.2	3.4-10.6		92	9.1	5.2-12.2	
extra cranial metastasis(-)	24	13.4	7.6-26.2	<0.01*	122	7.3	4.9-9.3	0.06
extra cranial metastasis(+)	40	3.7	2.8-6.2		111	3.9	3.3-5.2	
cyst(-)	45	6.6	3.5-8.3	0.68	161	4.7	3.7-7.2	0.29
cyst(+)	19	4.6	2.5-12.8		72	6.2	4.1-9.4	
posterior fossa lesion(-)	41	6.4	3.4-8.5	0.96	135	5.7	4.1-8.9	0.37
posterior fossa lesion(+)	23	6.3	2.5-12.8		98	4.7	3.4-6.9	
meningitis(-)	57	6.4	3.5-8.0	0.71	203	5.8	4.6-8.0	<0.01*
meningitis(+)	7	6.3	0.8-12.8		30	3.6	1.7-5.2	
hematoma(-)	63	6.4	3.5-8.0	0.56	226	5.2	4.1-7.4	0.32
hematoma(+)	1	4.3	NA-NA		7	3.6	0.4-9.4	
lung origin(-)	17	3.9	2.5-6.4	0.01*	94	4	3.3-5.2	0.01*
lung origin(+)	47	7.6	3.9-10.6		139	7.3	4.8-9.4	
breast origin(-)	62	6.4	3.5-8.0	0.89	203	5.2	4.0-7.3	0.45
breast origin(+)	2	8.5	4.3-NA		30	4.7	3.2-9.3	
digestive origin(-)	56	6.9	3.5-9.4	0.04*	200	5.7	4.4-7.9	0.01*
digestive origin(+)	8	4.3	0.1-6.4		33	3.7	1.3-5.2	
renal origin(-)	63	6.4	3.9-8.0	0.3	223	5.2	4.3-7.4	0.64
renal origin(+)	1	3.2	NA-NA		10	3.8	0.5-12.2	
others origin(-)	58	6.5	3.9-8.5	0.11	213	5.2	4.1-7.6	0.77
others origin(+)	6	2.7	0.9-NA		20	4.3	1.2-11.4	

KPS, Karnofsy performance status; mOS, median overall survival, *P<0.05.

**Table 3:** Multivariate analysis of median survival times for patients with synchronous and metachronous brain metastases.

Factor	HR	95% CI	P value
age ≧ 70	1.48	0.83-2.65	0.19
lung origin(+)	0.52	0.24-1.11	0.09
extra cranial metastasis(+)	1.97	1.09-3.56	0.03*
KPS ≧ 70	0.55	0.31-0.98	0.04*
digestive origin(+)	0.90	0.34-2.39	0.83

CI, confidence interval; HR, Hazard Ratio; KPS, Karnofsy performance status, * = P<0.05

### Verification of the proposed prognostic score in s-BM

Based on the above findings, we constructed a five-item score (age <70, KPS ≥70, no ECM, lung mass present, no digestive mass). Patients were stratified into three categories: Score A (0 points; n=6), Score B (1–4 points; n=31), and Score C (5–7 points; n=27) (**Table 4**).

**Table 4:** Prognostic score factors and classification criteria.

Factor	Evaluation criteria	Score
Digestive lesions	+	1
	−	0
Lung lesions	+	0
	−	1
Age	over 70	1
	under 70	0
extracranial metastasis	+	2
	−	0
KPS	under 60	2
	over 70	0

The total score is divided into three groups: 0 (A), 1 to 4 (B), and 5 to 7 (C).

KPS, Karnofsy performance status, score total range = 0–7.

**Figure 2:**
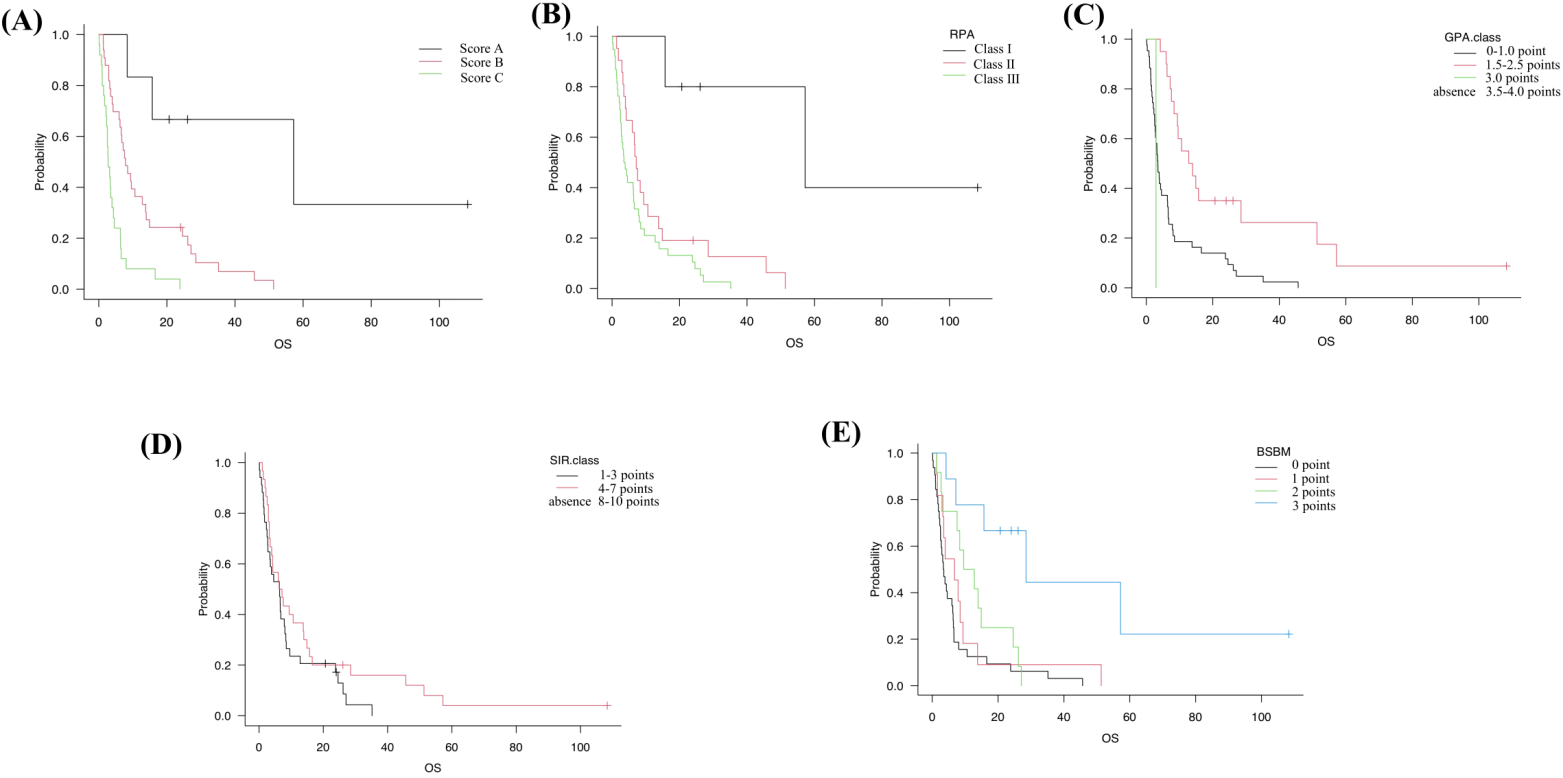
Kaplan–Meier OS in the s-BM cohort stratified by five prognostic systems. **(A)** Our score: Score A (0 points; n=6), Score B (1–4 points; n=31), Score C (5–7 points; n=27). **(B)** RPA (Recursive Partitioning Analysis): Class I (n=5), Class II (n=21), Class III (n=38). **(C)** GPA (Graded Prognostic Assessment): 0–1.0 (n=43), 1.5–2.5 (n=20), 3.0 (n=1). **(D)** SIR (Score Index for Radiosurgery): 1–3 (n=34), 4–7 (n=30). **(E)** BSBM (Basic Score for Brain Metastases): 0 (n=32), 1 (n=11), 2 (n=12), 3 (n=9). Tick marks indicate censoring; medians (with NE where appropriate) are reported in the text. Group differences were evaluated with two-sided log-rank tests across strata (trend interpretation for ordered categories); no multiplicity adjustment was applied, and pairwise p-values are not emphasized. These panels are provided for a clinical context; model-based performance comparisons are summarized in **Supplementary Tables S1**, **S2**.

mOS for Score A, Score B, and Score C was 57.2 months (95% CI, 8.3–NA), 7.6 months (95% CI, 4.2–12.8), and 3.2 months (95% CI, 2.1–4.6), respectively (**Figure 2A**), showing a clear stepwise gradient across categories.

### Comparison with established grading systems

For external context, the cohort was also classified by four established systems. RPA: Class I (n=5), Class II (n=21), Class III (n=38); GPA: 0–1.0 (n=43), 1.5–2.5 (n=20), 3.0 (n=1), 3.5–4.0 (n=0); SIR: 1–3 (n=34), 4–7 (n=30), 8–10 (n=0); BSBM: 0 (n=32), 1 (n=11), 2 (n=12), 3 (n=9).

Corresponding mOS values were as follows: RPA Class I, II, III: 57.2, 7.2, and 3.7 months (95% CI, 15.7–NA; 3.9–10.6; 2.5–6.4) (**Figure 2B**). GPA 0–1.0, 1.5–2.5, 3.0: 3.5, 13.4, and 2.9 months (95% CI, 2.5–6.3; 7.6–28.5; NA–NA) (**Figure 2C**). SIR 1–3 and 4–7: 6.4 and 6.7 months (95% CI, 2.7–8.0; 3.5–13.9) (**Figure 2D**). BSBM 0, 1, 2, 3: 3.4, 6.8, 11.2, and 28.5 months (95% CI, 2.3–6.2; 2.5–6.7; 2.7–24.6; 4.2–NA) (**Figure 2E**, **Table 5**). Among all systems, only our proposed score separated all categories distinctly across the cohort.

**Table 5:** Significant differences for each prognostic analysis score classification in this cohort.

BSBM	0point	1point	2points
1point	1.00	–	–
2points	0.49	1.00	–
3points	<0.01*	0.02*	0.04*
GPA	0-1.0point	1.5-2.5points	
1.5-2.5points	<0.01*	–	
3points	1	<0.01*	
RPA	I	II	
II	<0.01*	–	
III	<0.01*	0.15	
SIR	0-3points		
4-7points	0.15		
This study's score	a	b	
b	0.01*	–	
c	<0.01*	<0.01*	

BSBM, basic score for Brain Metastases; GPA, Graded Prognostic Assessment.

RPA, Recursive Partitioning Analysis; SIR, Score Index for Radiosurgery, *P<0.05.

There were no cases assigned to the GPA 3.5-4.0 points group or the SIR 8-10 points group in this cohort.

### Model-based performance in s-BM (C-index, AIC, PH)

**Figure 3:**
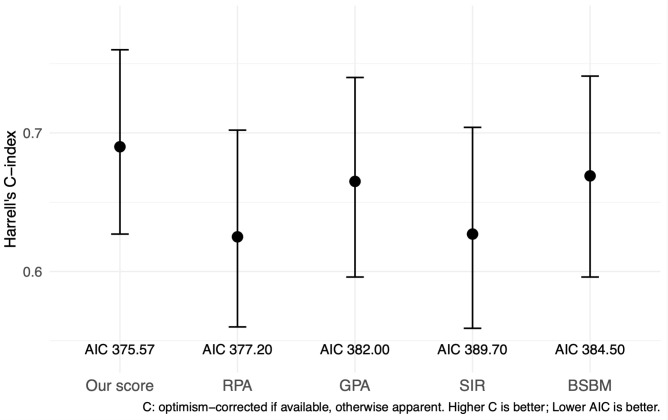
Harrell’s C-index with 95% confidence intervals for each prognostic scoring system in the s-BM cohort. AIC values are displayed below the labels. Higher C indicates better discrimination; lower AIC indicates better parsimony. Comparisons across systems are descriptive; no hypothesis testing was performed between C-indices. C-index, Harrell’s concordance index; AIC, Akaike information criterion; RPA, Recursive Partitioning Analysis; GPA, Graded Prognostic Assessment; SIR, Score Index for Radiosurgery; BSBM, Basic Score for Brain Metastases.

To complement Kaplan–Meier comparisons, we fitted univariable Cox models for each system in s-BM. Our score achieved the highest discrimination (Harrell’s C-index 0.690) and the lowest AIC (375.57) compared with RPA (C = 0.631/AIC=378.65), GPA (0.666/386.13), SIR (0.629/388.63), and BSBM (0.671/379.67). Global Schoenfeld tests indicated no PH violations for any model (our score p=0.983). (**Supplementary Table S1**) Global Schoenfeld tests did not indicate PH violations for our score (p = 0.983) or for the comparator models (all non-significant), supporting the validity of the Cox analyses. We summarized performance in **Table 6** (Harrell’s C-index, AIC, and 6/12-month AUCs). A compact one-panel plot (**Figure 3**) shows C-index point estimates across systems for visual comparison. In sensitivity analyses, the β-weighted variant achieved similar discrimination but a higher AIC, and the multivariable-only score did not outperform the 5-factor scheme; thus, we retained the pragmatic 1–2 point score for the main analyses (**Table 6**; **Supplementary Tables S1**, **S2**).

**Table 6:** Comparative prognostic performance of five scoring systems in the s-BM cohort—Harrell’s C-index, AIC, and time-dependent AUCs at 6 and 12 months.

Scoring system	Harrell’s C (apparent)	AIC	AUC 6 mo	AUC 12 mo
Our score	0.69	375.57	0.779	0.795
RPA	0.631	378.65	0.668	0.667
GPA	0.666	386.13	0.729	0.706
SIR	0.629	388.63	0.662	0.702
BSBM	0.671	379.67	0.711	0.782

C-index: Harrell; bootstrap internal validation (B=500) used elsewhere in the manuscript; optimism-corrected C for our score ≈ 0.691.

AUC: time-dependent AUC (timeROC); for categorical systems, the Cox linear predictor was used as the marker.

RPA, Recursive Partitioning Analysis; GPA, Graded Prognostic Assessment; SIR, Score Index for Radiosurgery.

BSBM, Basic Score for Brain Metastases; C-index, Harrell’s concordance index; AIC, Akaike information criterion; AUC.

### Time-dependent AUCs at 6 and 12 months

Time-dependent AUCs favored our score at both landmarks: 0.779 (6 months) and 0.795 (12 months), versus RPA 0.668/0.667, GPA 0.729/0.706, SIR 0.662/0.702, and BSBM 0.711/0.782 (categorical scores evaluated using Cox linear predictors). (**Supplementary Table S1**).

### Internal validation and sensitivity analysis

Bootstrap internal validation (B = 500) yielded an optimism-corrected C-index of approximately 0.691 for our score, closely matching the apparent estimate, suggesting minimal optimism. Comparator systems had corrected C-indices of ~0.625–0.671.

In a sensitivity analysis, a coefficient-weighted variant of our score (multivariable Cox linear predictor) showed C-index 0.694 and AIC 381.9—comparable discrimination but worse parsimony than the simple scheme. An integer approximation of the weighted model is provided in the Supplement. (**Supplementary Tables S1**, **S2**).

## Discussion

Previously, it has been considered that the prognosis of BM was dependent on tumor size in terms of sensitivity to radiation therapy, which was supposed to play a pivotal role in the treatment of BM.

However, prognostic stratification for clinical outcomes following the introduction of molecular targeted drugs and ICIs has become more evident than prognostication after radiation therapy, therefore the credibility of traditional prognosis grading has become questionable.

The concept of chemo-immunotherapy differs significantly from radiation therapy; specifically, its effectiveness is determined by sensitivity rather than tumor size.

Furthermore, intracranial irradiation is a double-edged treatment that is beneficial due to its cytotoxic effect from radiation therapy, but it also induces cerebral edema and radiation necrosis. Although steroids can help alleviate cerebral edema, they also suppress the immune system.

Instead, VEGF inhibitors like bevacizumab have been introduced. They provide beneficial dual effects by reducing cerebral edema and enhancing tumor immunity. Therefore, with the advancement of these multimodalities for BM, the traditional grading system must be reevaluated for accurate prognostication.

So far, the GPA scoring system for BM has been revised, incorporating genetic backgrounds over time (2, 6–8). Accurate prognostic predictions made during the initial consultation, as analyzed in this study, are essential for further treatment.

In this cohort, overall survival appeared similar between s-BM and m-BM; however, this two-group comparison is exploratory and not powered for equivalence/non-inferiority. Historically, s-BM has been reported to fare worse (9, 10). Differences in case-mix and treatment exposure likely counterbalanced (e.g., s-BM: more lung, fewer breast, more surgery, and less conventional chemotherapy; m-BM: exclusion of early failures and intracranial therapy prioritized once primary treatment is established) (11), and contemporary systemic therapies may further attenuate historical gaps (12). Accordingly, we emphasize the development of an s-BM–specific, pre-treatment score usable at the initial consultation.

In the background analysis of groups of s-BM and m-BM of this cohort, there were more lung cancers in groups of s-BM and more breast cancers in groups of m-BM, which is consistent with previous reports, including the characteristic that breast cancer often metastasizes to other organs after a while. The higher number of surgical interventions in the groups of s-BM is presumably due to the high need for surgical treatment to improve symptomatic cases and the need for tissue confirmation. Potthoff et al. stated that there was no difference in prognosis after surgical intervention between s-BM and m-BM, thus surgical treatment should be preferentially considered (13).

Previous reports have shown that prognosis was dependent on primary cancer, especially digestive cancer, being particularly associated with a poor prognosis (14). Interestingly, similar results were observed in s-BM in this cohort.

There are several reports summarizing the treatment outcomes of the group of s-BM by primary cancer type, and it has been reported that age, tissue type, systemic treatment, and surgical treatment are involved as prognostic factors in BM from breast cancer and lung cancer (15–18).

Our score demonstrated the highest discrimination (C-index 0.690) and the lowest AIC (375.57) compared with RPA, GPA, SIR, and BSBM, with no PH violations; time-dependent AUCs at 6/12 months were concordant, and bootstrap-corrected C-indices were nearly identical to apparent estimates—suggesting limited optimism. A coefficient-weighted variant achieved similar discrimination but a higher AIC, supporting the pragmatic 1–2 point scheme for time-zero use. Variable selection based solely on multivariable significance can be sample-size dependent and unstable in correlated predictors, risking the omission of clinically salient time-zero information. In our data, a β-weighted score and a multivariable-only score offered no material performance gain over the simple scheme, while reducing usability; hence the final model prioritizes parsimony and interpretability with internal bootstrap validation.

When considering the treatment outcomes of BM, the influence of racial differences could not be ignored because of the wide variety of primary tumors. The proportion of melanoma is higher in cohorts that include Caucasians, while the proportion of gastrointestinal and thyroid cancers tends to be higher in cohorts that include Asians (19, 20). Among these, the results of this cohort are an analysis of a single institution, but they are likely to reflect the treatment outcomes of patients with BM in Japan.

The score we proposed here is intended to predict the prognosis at the time of initial consultation in cases where both conditions are discovered synchronously. As mentioned above, this score is capable of more sensitive classification than other scores, but to assess the usefulness of this score, it is necessary to take into account the unique characteristics of BM treatment. Naturally, unlike other intracranial BM, treatment must be carried out in coordination with the treatment of the primary lesion. Unlike the group of m-BM, in which metastasis was discovered after the diagnosis was confirmed and treatment had progressed, in the group of s-BM, collaboration with other departments is particularly necessary to determine whether tissue confirmation should be performed at the primary or metastatic lesion, and how the treatment should be scheduled. We also formulate treatment schedules in consultation with the department in charge of the primary tumor, and it is important to be able to make highly accurate prognostic predictions at this time. Based on this, a common understanding can be reached as to whether aggressive treatment should be recommended or palliative treatment should be considered, and we hope that our proposal scoring system will be utilized appropriately in the future to promote collaboration.

### Limitation

This study was conducted at a single center and retrospectively analyzed. Although bootstrap internal validation (B = 500) suggested minimal optimism, the score has not yet undergone external validation. All modeling and performance estimates were derived from the same institution and period, so generalizability across centers, imaging protocols, disease mix, and systemic therapy patterns remains uncertain. Given the modest size of the s-BM cohort (n=64; ~60 events) and the use of the same cohort for both model development and validation, a non-negligible risk of overfitting remains. We prespecified the five predictors and their cut-offs and avoided data-driven stepwise selection to mitigate this risk, and the optimism-corrected C-index (0.691) was close to the apparent estimate; nevertheless, residual optimism cannot be excluded. A limitation of this time-zero design is possible misclassification of the primary site when using imaging surrogates; such an error is likely non-differential and would attenuate associations. External, multicenter validation with standardized imaging adjudication and, where available, pathology cross-check is warranted. In line with reporting guidance for prediction models, the present work represents model development with internal validation; external, multicenter—preferably prospective—validation and potential recalibration are required before broad implementation. We plan such validation in an independent, multicenter s-BM cohort.

Finally, systemic therapy has evolved rapidly in recent years (e.g., immune checkpoint inhibitors and targeted agents). Our development cohort spans both the pre-immunotherapy era and its early adoption. However, the score was based on regimen-agnostic pretreatment factors; its performance may differ in a setting dominated by immunotherapy. Given the limited number of patients treated exclusively with immunotherapy in our s-BM cohort, we did not perform a restricted analysis for this subgroup. This limitation will be addressed in future external, multicenter validation. Treatment-era heterogeneity remains essential, and we plan to pursue an immunotherapy-only subanalysis in a contemporary, multicenter cohort.

## Data Availability

The raw data supporting the conclusions of this article will be made available by the authors, without undue reservation.
